# Peculiarities of the Edaphic Cyanobacterium *Nostoc linckia* Culture Response and Heavy Metal Accumulation from Copper-Containing Multimetal Systems

**DOI:** 10.3390/toxics10030113

**Published:** 2022-02-27

**Authors:** Liliana Cepoi, Inga Zinicovscaia, Ana Valuta, Liviu Codreanu, Ludmila Rudi, Tatiana Chiriac, Nikita Yushin, Dmitrii Grozdov, Alexandra Peshkova

**Affiliations:** 1 Institute of Microbiology and Biotechnology, 1, Academiei Str., 2028MD Chisinau, Moldova; lilianacepoi@yahoo.com (L.C.); annavaluta@yahoo.com (A.V.); co_liv@mail.ru (L.C.); rudiludmila@gmail.com (L.R.); chiriac_tv@yahoo.com (T.C.); 2 Joint Institute for Nuclear Research, 6 Joliot-Curie Str., 1419890 Dubna, Russia; ynik_62@mail.ru (N.Y.); dsgrozdov@rambler.ru (D.G.); peshkova.alexandra92@gmail.com (A.P.); 3 Department of Nuclear Physics, Horia Hulubei National Institute for R&D in Physics and Nuclear Engineering, 30 Reactorului Str. MG-6, 077125 Magurele, Romania

**Keywords:** *Nostoc linckia*, bioaccumulation, copper, multimetal contamination, biochemical modification, neutron activation analysis

## Abstract

Soil and water pollution is a major problem that has a negative impact on ecosystems and human health in particular. In the bioremediation processes, the application of photosynthetic microorganisms, including cyanobacteria, is a direction of action addressed with increasing frequency in the context of further development and improvement of environmentally friendly techniques needed for detoxification of soils and waters polluted with low concentrations of toxic elements, since they pose a challenge for traditional treatment methods. In the present study, the removal of copper and other metal ions from multielement systems by three generations of *Nostoc linckia* is discussed. Changes in the biochemical composition of the nostoc biomass, which accumulates metal ions, were monitored. Neutron activation analysis was applied to assess Cu, Fe, Ni, and Zn accumulation by biomass, as well as to determine the biochemical composition of biomass after specific biochemical methods were used. The capacity of the accumulation of copper and other metal ions from multi-elemental systems by cyanobacteria *Nostoc linckia* was high and increased over two cycles of biomass growth in the systems Cu-Fe-Ni and Cu-Fe-Zn and over three cycles in Cu-Fe and Cu-Fe-Ni-Zn systems. It constituted 1720–10,600 µg metal/g depending on the system and cycle of cultivation. The accumulation of Fe, Ni, and Zn also increased over the generations of nostoc. The process of metal accumulation was demonstrated by a significant change in the biomass biochemical composition. Cyanobacteria *Nostoc linckia* possess a pronounced capacity of copper and other metal ion accumulation from multimetal systems and showed an increased resistance in environments polluted with heavy metals.

## 1. Introduction

Soil and water pollution with heavy metals is a major problem for the health of natural and man-made ecosystems. Several heavy metals in certain quantities are essential for the biological activity of various living organisms, being a part of the key enzymes involved in the realization of vital biochemical reactions. In quantities that exceed tolerance limits, metals become extremely toxic, affecting almost all vital processes in cells.

Copper is one of the elements with a dual effect on living organisms. On the one hand, copper is strictly needed for the majority of living organisms. Its deficiency can provoke anemia caused by disorders of hematopoietic processes, dysfunction of the ophthalmic nerve, neuropathy and ataxia—effects primarily resulting from a decrease of the activity of copper-dependent enzymes [1,2]. 

Photosynthetic organisms can also be affected by a deficiency of copper, which is manifested by disturbances of chlorophyll, protein, and carbohydrate synthesis, and of the photosynthesis process, as well as the disruption of electron transfer in the respiratory chain [3]. On the other hand, copper excess can seriously damage organisms. For example, in the human body, a high dose of copper can lead to severe pathological disruption in the liver and neurodegenerative diseases, including Alzheimer’s disease [4].

The distribution of copper (Cu) in the soil is very diverse depending on the geographic location, climatic and geological factors, and physicochemical properties of the soil, such as the carbon content, pH, and soil texture. The level of copper in the soil is influenced by industrial pollution and agricultural activity—especially the growth of perennial crops. 

A recent study conducted on multiple soil sites in European countries showed that the average copper concentration over the studied regions was 16.85 mg/kg soil (with a range of 0 to 496.3 mg/kg), while the soils of vineyards, olive gardens, and orchards contained 27.3–49.3 mg Cu per kg of soil [4]. This pollution is caused by the use of copper-containing fungicides for treating vines and orchards. According to the Government Decree on the Assessment of Soil Contamination and Remediation Needs 214/2007, the copper threshold value is 100 mg kg^−1^, and the guideline value is 150 mg kg^−1^ [4]. 

In addition to copper, soils contain other metal ions, such as iron, nickel, zinc, etc., which are required for the growth and vitality of living organisms but excessive amounts of these metals can become toxic. Thus, zinc is a constituent of many proteins, it is also an enzyme cofactor and is fundamental for optimum plant growth and development. However, at high concentrations in the soil, Zn is phytotoxic, and plants that accumulate it through root absorption or deposition pose a health risk to consumers [5]. Nickel is an essential micronutrient required for the growth of higher plants. 

It has been established that nickel from anthropogenic sources is more readily taken up by plants than from naturally occurring sources, and that plant species also differ in their tolerance and ability to take up nickel from soils [6]. Iron represents the most common element on Earth and the most frequently utilized transition metal in the biosphere. Iron is essential for the production of hemoglobin, myoglobin, and several essential enzymes and is involved in DNA synthesis. Chronic inhalation of excessive concentrations of iron oxide dust may result in the development of benign pneumoconiosis [7].

In order to remediate soils contaminated with copper and other metal ions, several groups of physical, chemical, and biological methods are taken into account. Their application depends on the working limits of the processes (concentrations of pollutants, co-presence of other metals or organic waste, etc.). Thus, physical methods are applicable for severely polluted sites and are very laborious. Chemical methods are currently very popular, especially due to their effectiveness and speed, while bioremediation is the most popular and intensely researched method due to its environmentally friendly nature and high efficiency [3,8]. Bioremediation using plants and microorganisms can be applied over large areas and are suitable even at low levels of metal content in the soil.

Microorganisms can remove copper from the soil via passive mechanisms, such as biosorption, as well as active mechanisms, such as the bioaccumulation and secretion of siderophores. Bacterial monocultures, such as *Pseudomonas stutzeri*, *Stenotrophomonas maltophilia*, etc. [9,10] and consortia of sulfur-reducing bacteria [11], root nodule bacteria [12], and metal resistant bacteria isolated from polluted sites [13] can all be used in the process of copper removal from polluted sites. Less studied in this respect are soil cyanobacteria. 

These organisms are the first colonizers of soil ecosystems and are a part of the biological soil crusts in the surface layers. The functions of these organisms in the soil ecosystem are different, the main ones being the fixation of nitrogen and carbon. Soil cyanobacteria also increase soil fertility by synthesizing and releasing organic polymers. 

Additionally, the release of exopolysaccharides into the soil improves soil stability [14]. In recent years, it has been proposed to use cyanobacteria as a biotechnological tool for the restoration of soil ecosystems—in particular, salt-affected ones [15]. 

The efficiencies of copper, cobalt, lead, and manganese accumulation by the cyanobacteria *Nostoc muscorum* and *Anabaena subcylindrica* were found to be 12.5–81.8%, 11.8–33.7%, 26.4–100%, and 32.7–100%, respectively [16]. *Nostoc muscorum*, a native cyanobacterial species isolated from a coal mining site, was employed to remove Cu(II), Zn(II), Pb(II), and Cd(II) from an aqueous solution containing these metals in the mixture. The results revealed a maximum removal of both Pb(II) (96.3%) and Cu(II) (96.42%) followed by Cd(II) (80.04%) and Zn(II) (71.3%) at the end of a 60-h culture period [17]. *Nostoc muscorum* was able to accumulate 66% of Zn(II) and 71% of Cu(II) within 24 h of contact time [18].

The presence of multiple functional groups on the surface of the polysaccharide capsules of soil cyanobacteria can be useful for binding metals, which enter soil and water in different ways. *Nostoc linckia* is a soil cyanobacterial species that also grow in the aquatic environment. The species is known for its ability to remove various metals, including copper, from single and multi-metal systems [19,20]. These properties are useful in the process of identifying bioremediating organisms, which can be used at various types of contaminated sites, for example in water and on the surface of the soil, as in the case of nostoc. 

Cyanobacteria, including *Nostoc*, are very sensitive to the presence of copper in the environment; therefore, this element is present in compounds with an algicidal effect that prevents water bloom. However, at low concentrations of copper (0.5 mg/L), nostoc biomass can accumulate amounts of this metal that exceed those under normal conditions by more than 20 times [19]. The metals from the studied systems, including copper, essentially influence both the growth rate of cyanobacterial cultures and the quality of the obtained biomass. Among the most common effects are a reduction of the growth rate, decrease of protein and pigment content in the biomass, fluctuation of carbohydrate content depending on the concentration of metals, increased oxidative stress markers, and altered antioxidant activity of the biomass [19,21,22].

All of the abovementioned effects/parameters have been studied in a single cycle of cultivation of cyanobacteria, while in reality, pollutions have a recurrent or permanent character. Thus, it is important to investigate both the bioaccumulation capacity of cyanobacteria to multiple interactions with different pollutants and their ability to adapt to the conditions of long-term pollutant exposure.

The first step in identifying bioremediators, both for soil and water purification, is to conduct experiments in model systems that can accurately quantify the performance of organisms to remove metals and other pollutants. In the case of *Nostoc linckia*, the second stage can be performed in real contaminated aquatic environments and soil conditions. The existing technologies for soil colonization with *Nostoc linckia*, the ability of this species to grow actively in the upper layers of the soil and to form crusts on the surface, its drought resistance properties and minimum requirements for survival support the possibility of successfully using this cyanobacterium to remedy both water and soil.

The scope of the present study is to highlight the effects of the repeated action of copper in combination with other metal ions on the accumulation of biomass and the biochemical composition of *Nostoc linckia*, as well as to assess the bioaccumulation capacity of biomass under experimental conditions in the model aquatic system.

## 2. Materials and Methods

**Figure 1:**
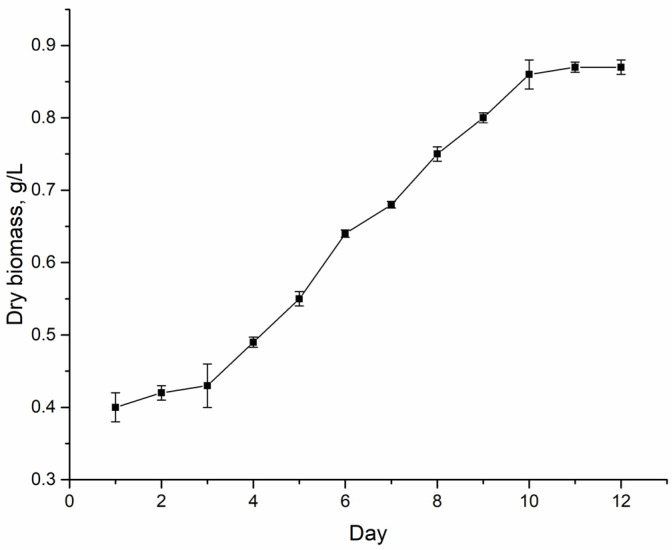
Growth curve of *Nostoc linckia* (Roth) Born et Flah CNM-CB-03 in standard conditions.

The experiments were performed using the cyanobacterial strain *Nostoc linckia* (Roth) Born et Flah CNM-CB-03, deposited in the National Collection of Nonpathogenic Microorganisms of the Republic of Moldova. The growth cycle of this strain in a closed system with a nitrogen-containing medium is 12 days. The exponential growth phase lasts from the 4th to the 10th day (Figure 1).

For obtaining inoculum for the first cycle cyanobacteria was grown in a medium containing: *macroelements* (g/L)—KNO_3_—0.5, K_2_HPO_4_—0.45, NaHCO_3_—0.05, MgSO_4_·7H_2_O—0.1, CaCl_2_—0.11 and *microelements* (mg/L)—ZnSO_4_·7H_2_O—0.05, MnSO_4_—2.0, H_3_BO_3_—0.85, (NH_4_)_6_Mo_7_O_24_·4H_2_O—2.25, FeSO_4_ 7H_2_O—4.0, Co(NO_3_)_2_·H_2_O—0.009, and EDTA—4.75. The same medium was used in all experiments but without microelements present in the experimental systems. 

Cyanobacteria cultivation was conducted in Erlenmeyer flasks of 1000 mL with a working volume of 0.5 L. The following parameters of the cultivation process in laboratory conditions were maintained: pH of the medium 6.8–7.2, temperature 25–27 °C, continuous illumination with a light intensity of 37–55 μmol photons/m^2^/s, and slow periodic shaking. The amount of inoculum was 0.4 g/L (in terms of dry biomass).

Metals were added to the culture medium on the sixth day of biomass growth (exponential growth phase) when the culture is more sensitive to external actions, including heavy metals [21,23]. The following salts were used for multimetal batch system preparation: CuSO_4_,·FeCl_3_·6H_2_O, Ni(NO_3_)_2_·6H_2_O, and ZnCl_2_ (purity ≥ 97%) (Sigma-Aldrich, Darmstadt, Germany). 

Four multielement systems were modeled, which were noted as follows: Cu/Fe, Cu/Fe/Ni, Cu/Fe/Zn, and Cu/Fe/Ni/Zn, and their chemical composition is given in Table 1. The selection of metal concentrations was determined by the ability of nostoc biomass to survive at given values in multielement systems. 

The cultivation cycle lasted 12 days, and the pH of the mixture during one cultivation cycle was in the range of 6.8–7.2. At the end of the cultivation cycle, biomass was separated from the cultivation medium by centrifugation and repeatedly washed with distilled water to remove the remaining salts. Biomass from each flask was divided into three portions: one portion was used for biochemical analysis, the second for neutron activation analysis, and the third for subculturing. The procedure was repeated at the end of the second and third cycles of biomass cultivation. Subculturing was conducted strictly within the limits of the same experimental variant so that the conditions in the second and third cultivation cycles were identical to the first. 

Subcultivation from the control sample was done in the same way—the biomass was transferred to a fresh control medium for the second and third cycles. Raw biomass for analysis was standardized to a concentration of 10 mg of dry biomass in 1 mL of suspension, with distilled water. Hereinafter all the values for biomass are expressed as dry biomass. For all biochemical tests, biomass was subjected to a repeated freeze-thaw procedure. The biomass used for neutron activation analysis was dried at 105 ± 2 °C to a constant weight. The same procedure was performed with control biomass but without the addition of metal ions.

### 2.1. Biochemical Analysis

The protein content in samples of 10 mg/mL of biomass was determined spectrophotometrically by the Lowry method. The biomass samples were processed with 0.1 N NaOH solution for 30 min at room temperature (0.1 mL of biomass suspension + 0.9 mL of 0.1 N NaOH). Then, to 0.2 mL protein extract, we added 2.0 mL reagent consisting of 49 mL of 2% sodium carbonate in 0.1 N sodium hydroxide and 1.0 mL of 0.5% copper sulfate in 1.0% sodium citrate. 0.2 mL of Folin–Ciocalteu reagent diluted four times was added to the reaction mixture. Samples were subjected to incubation for 30 min at room temperature, after which the absorbance was measured at 750 nm. Protein content was calculated using a calibration curve for bovine serum albumin [24].

The carbohydrate content was determined using the Anthrone reagent. The reaction mixture was composed of 0.25 mL of analyzed sample from 10 mg/mL biomass and 2.5 mL of 0.5% anthrone solution in 66% sulphuric acid. The obtained samples were subjected to boiling at a water bath for 30 min, followed by a period of incubation in the dark for 30 min. The sample absorbance was measured at 620 nm. The carbohydrate content was calculated using a calibration curve for glucose [25].

Quantitative determination of lipids was conducted using the phospho-vanillin reagent. To obtain lipidic extract to 10 mg of biomass was added 1 mL of a mixture of chloroform and ethanol in the ratio of 9:1 (*v:v*). Extraction was performed at room temperature by continuous stirring for 120 min. The lipidic extract was subjected to hydrolysis with sulfuric acid (66%) and 0.1 mL of hydrolysate was mixed with 2.9 mL of phospho-vanillin reagent. The color of the samples developed for 30 min, after which the absorbance was measured at the wavelength of 560 nm. The lipid content was calculated using a calibration curve based on oleic acid [26].

Phycobiliprotein content was calculated based on the formula proposed by Siegelman and Kycia [27]. The liquid fraction of 3 mL of standardized freeze-thawed biomass was separated by centrifugation and the absorbance was measured at 565 nm for phycoerythrin, 620 nm for phycocyanin, and 650 nm for allophycocyanin.

The content of chlorophyll and β-carotene in biomass was determined on the basis of ethanolic extracts. A mixture of 10 mg of biomass with 1.0 mL of 96% ethanol was prepared. Pigment extraction was performed by continuous stirring at room temperature for 12 h. The ethanolic extract was separated from biomass by centrifugation. The chlorophyll content was determined based on the absorbance at 665 nm and extinction coefficient of 0.8 × 10^5^ M^−1^·cm^−1^, and β-carotene—based on the absorbance at 450 nm and extinction coefficient 1.5 × 10^5^ M^−1^·cm^−1^ [25].

Determination of the content of malondialdehyde (MDA) in biomass is based on the reactive substances of thiobarbituric acid (TBA). To 10 mg of biomass were added 3.0 mL of 0.76% solution of TBA in a 20% solution of trichloroacetic acid. The reaction mixture was incubated at 95 °C for 20 min, then samples were cooled and the optical density was measured at wavelengths of 532 nm and 600 nm. The amount of MDA in the samples was calculated using the molar extinction coefficient for the MDA-TBA complex [28].

The antioxidant activity was established using ABTS (2,2′-azino-bis(3-ethylbenzothiazoline-6-sulfonic acid) assay [29]. Ethanolic extract was obtained in the same way as the extract for pigment determination. The radical cation ABTS ^+^ is produced from the oxidation with potassium persulfate. ABTS stock solution was prepared by mixing 7 mM of ABTS in deionized water with 2.45 mM potassium persulfate in a ratio of 1:1 *(v:v*). The oxidation of ABTS occurred in the dark at room temperature for at least 12–16 h. The working solution of ABTS had an absorbance of 0.700 ± 0.020 at 734 nm. The samples were obtained by mixing 0.3 mL of ethanolic extract and 2.7 mL of ABTS solution. The absorbance of the samples was measured after 6 min, and the % inhibition relative to the absorbance of the ABTS reagent was calculated.

### 2.2. Neutron Activation Analysis

The content of elements accumulated by the *Nostoc linckia* biomass was determined by instrumental neutron activation analysis (NAA) at the REGATA facility of the IBR-2 reactor (Dubna, Russia) The procedure of biological sample irradiation can be found elsewhere [25,30,31]. To determine the content of Fe, Ni, and Zn in biomass, the samples were packed in aluminum cups and irradiated for 3 days at a neutron flux of 1.2 × 10^11^ n cm^−2^ s^−1^, re-packed, and measured twice using HP germanium detectors after 4 and 20 days of decay, respectively. 

The iron content was determined by a *γ*-line with energy of 1099.25 keV of the isotope ^59^Fe, nickel by a *γ*-line with energy of 810.57 keV of the isotope ^58^Co, and zinc by a *γ*-line with energy of 1115.54 keV of the isotope ^65^Zn. To determine copper content, the samples were packed in polyethylene bags, irradiated for 3 min at a neutron flux of 1.1 × 10^12^ n cm^−2^ s^−1,^ and measured immediately after irradiation. The copper content in the samples was determined by a *γ*-line with energy of 1039.2 keV of the isotope ^66^Cu. NAA data processing was done using the software Genie 2000 and calculation of element concentrations was performed by a relative method using the software developed at the Frank Laboratory of Neutron Physics, JINR.

### 2.3. Statistical Analysis 

All experiments were performed in triplicate. The results in all histograms are presented as the mean values ± standard deviations. Differences between the values were estimated by Student’s t-tests. The following comparisons were taken into account: (1) each experimental variant with control; (2) cycle I with cycle II; (3) cycle I with cycle III; and (4) cycle II with cycle III.

## 3. Results

### 3.1. Biomass Obtained at the End of the Three Cycles of Nostoc Growth 

**Figure 2:**
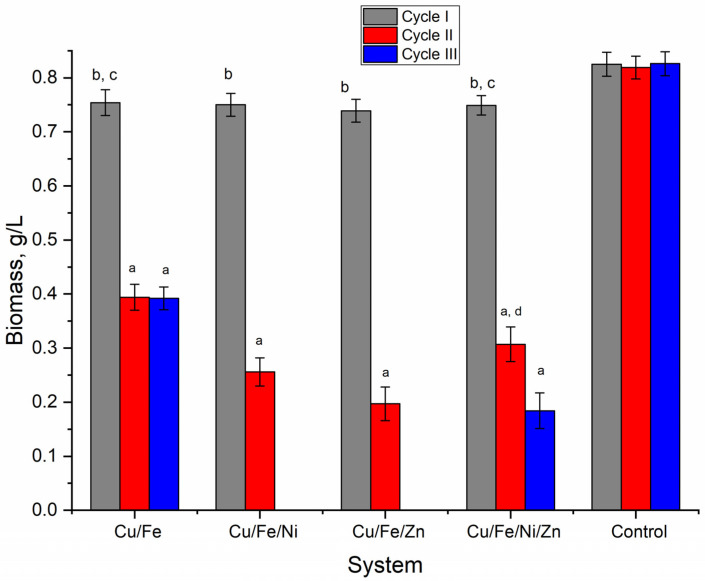
Nostoc linckia dry biomass accumulated at the end of three cultivation cycles in multimetal systems (a—*p* < 0.001 for the difference between experimental and control samples, b—*p* < 0.001 for the difference between the first and second cycle, c—*p* < 0.001 for the difference between the first and third cycles, and d—*p* < 0.001 for the difference between the second and third cycles).

Nostoc biomass was determined at the end of each of the three cultivation cycles (Figure 2). The biomass of control samples in all three cultivation cycles was between 0.819 and 0.825 g/L. In experimental variants at the end of the first cultivation cycle, the biomass quantity, 0.739–0.754 g/L, was very similar in all studied multimetal systems and constituted 89.4–91.4% of the control biomass. The reduction of the amount of biomass was moderate and less pronounced. The situation changed drastically in the second cultivation cycle. 

As inoculum in this cycle, the biomass obtained at the end of the first cycle was used. In the second cycle, the biomass accumulated at the end was between 0.197 and 0.394 g/L, which is 2.1–4.2-times less compared to the control (*p* < 0.001). The highest level of biomass in the second cycle was obtained in the bimetallic Cu/Fe system (0.394 g/L), followed by the four-component system Cu/Fe/Ni/Zn (0.307 g/L), and three-component systems, Cu/Fe/Ni and Cu/Fe/Zn, with 0.256 and 0.197 g/L, respectively.

The third cultivation cycle was fatal for the nostoc culture in two of the four studied systems—Cu/Fe/Ni and Cu/Fe/Zn, in which autolysis took place. In the other two systems that ensured the survival of the nostoc culture in the third cultivation cycle, the situation was different. Thus, in the Cu/Fe system, the amount of accumulated biomass was at the level of the second cycle, and in the Cu/Fe/Ni/Zn system, the biomass accumulation continued to decrease, as it was lesser by 40% compared to the second cycle and by 78% compared to the first cycle. Thus, copper-containing multimetal systems led to a non-essential reduction in the nostoc productivity in the first cultivation cycle but significantly affected it in subsequent cycles, resulting in a significant decrease in biomass or even the death of the culture.

### 3.2. Copper and Other Metal Ion Accumulation in Nostoc Biomass from Multielement Systems 

**Figure 3:**
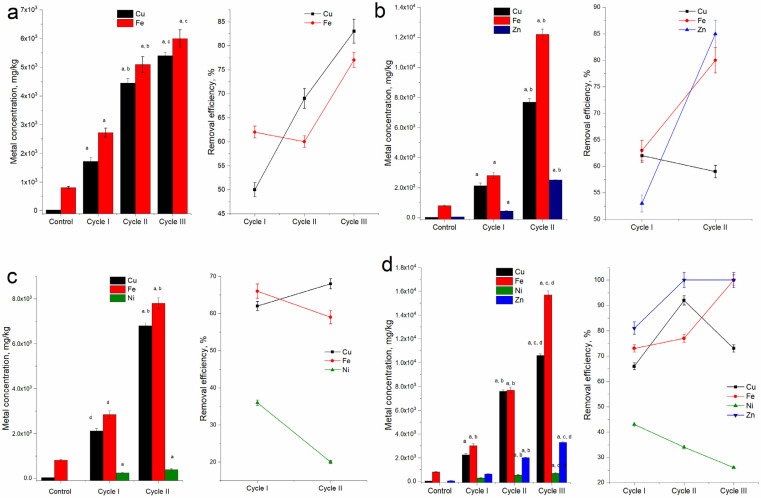
Bioaccumulation of copper and other metals by *Nostoc linckia* biomass during three life cycles in media containing multimetallic systems (**a**) Cu/Fe, (**b**) Cu/Fe/Zn, (**c**) Cu/Fe/Ni and (**d**) Cu/Fe/Ni/Zn (a—*p* < 0.001 for the difference between experimental and control samples, b—*p* < 0.001 for the difference between the first and second cycle, c—*p* < 0.001 for the difference between the first and third cycles, and d—*p* < 0.001 for the difference between the second and third cycles).

The results related to the accumulation of metals in the nostoc biomass in four polymetallic systems during three cycles of cultivation are presented in Figure 3. The amount of copper in nostoc biomass in the control was 32 mg in per kilogram of dry biomass. In the presence of the metal Cu in the nutrient medium, its amount in biomass increased impressively. Depending on the analyzed system and the cultivation cycle, the amount varied from 1720 to 10,600 mg Cu/kg biomass. In the Cu/Fe system in the first cultivation cycle, the biomass accumulated 1720 mg/kg of copper, which constituted approximately 50% of the copper present in the system. 

In the second cycle, the amount of copper in per kilogram of biomass increased 2.6 times compared to the first cycle and was 4450 mg (*p* < 0.001). The increase in copper accumulation also took place during the third cultivation cycle. Thus, at the end of the third cycle, the amount of copper in per kilogram of the nostoc biomass was 5400 mg, significantly higher than in the second cycle (*p* < 0.001). This value, in relation to the level of productivity and the copper content in the system, constituted 84% of the copper introduced into the system.

In the Cu/Fe/Ni system at the end of the first cultivation cycle, the biomass accumulated 2120 mg/kg of copper. Compared to the amount of biomass, the level of copper removal from the system in the first cycle was 51.9%. In the second cultivation cycle, the amount of copper accumulated in per kilogram of biomass was 6800 mg, which corresponded to the removal of 70% of copper ions from the system. 

The third Cu/Fe/Zn system behaved very similarly to the previous one, as the nostoc survived during the first two cycles of cultivation. Thus, at the end of the first cycle, the amount of copper in per kilogram of biomass was 2130 mg, corresponding to the removal of 62% of the copper present in the system. At the end of the second cycle, the amount of copper in per kilogram of biomass increased 3.6 times compared to the first cycle and constituted 7700 mg; however, due to the drastic reduction in the amount of biomass, the efficiency of copper removal was on the level of the first cycle. 

In the fourth system (Cu/Fe/Ni/Zn), nostoc accumulated copper the most efficiently. Thus, at the end of the first cultivation cycle, the amount of copper in per kilogram of biomass was 2240 mg, which corresponded to the removal of 67% of the copper present in the system. In the second cycle of cultivation, the metal amount in per kilogram of biomass reached the value of 7600 mg and it was 3.4-times higher than in the first cycle. At this point was the highest efficiency of copper ion removal, at 93% of the metal ions introduced into the system. At the end of the third cultivation cycle, the highest copper accumulation in biomass was attained—10,600 mg/kg; however, due to the considerable reduction in the amount of biomass, the efficiency of removal decreased to the level of the first cycle.

The biomass accumulated a significant amount of iron in all studied systems, and as in the case of copper, the accumulation increased over the cycles. In the control biomass, the amount of Fe in per kilogram was 810 mg, while in the experimental variants, it reached values from 2720 to 15,700 mg/kg, which was significantly higher (3.4–19.4 times), compared to the control (*p* < 0.001). Although the iron content in biomass increased over the cycles, the efficiency of its removal from the analyzed systems remained almost at the same level, with small exceptions. 

This was mainly due to the significant decrease in the amount of biomass. In the Cu/Fe system, the amount of iron in per kilogram of biomass in the three cycles were 2720, 5090, and 6000 mg, respectively, and the efficiencies of its removal from solution were 62%, 60%, and 67%. In the Cu/Fe/Ni system at the end of the first cultivation cycle, the nostoc accumulated 2850 mg/kg of iron, and at the end of the second—7800 mg/kg (2.7-times more). The efficiency of iron removal in the two cycles was 66.4% and 59.3%, respectively, which is explained by the decrease in biomass quantity in the second cycle.

Similarly, in the Cu/Fe/Zn system in the first cycle, nostoc biomass accumulated 2810 mg/kg of iron and in the second—12,200 mg/kg (4.3-times more), while the efficiency of iron removal increased from 63.3% to 79.7%. In the last studied system (Cu/Fe/Ni/Zn), nostoc biomass accumulated iron the most efficiently. The amount of metal in per kilogram of biomass increased over the cycles and constituted 3030, 7700, and 15,700 mg, the efficiency of iron ion removal also increased from 73% in the first cycle to practically 100% in the third. However, it should be noted that the amount of biomass in the second, and especially in the third cultivation cycle, was significantly reduced.

Nickel content in control biomass was below the detection limit of NAA, and in the two systems containing it, its accumulation took place. Thus, at the end of the first cycle, its amount in the Cu/Fe/Ni system in per kilogram was 244 mg and 287 mg in the Cu/Fe/Ni/Zn system. At the end of the second cultivation cycle, the amount of accumulated nickel from the two systems increased 1.6 and 1.9 times, respectively, constituting 390 and 550 mg in per kilogram. In the third cycle, the nostoc culture survived only in the Cu/Fe/Ni/Zn system, in which the amount of nickel accumulated in the biomass increased again by a factor of 1.3 compared to the second cycle. In comparison with copper and iron, the accumulation of nickel was a less efficient process, its maximum removal was 42% in the first cycle in the Cu/Fe/Ni/Zn system.

The amount of zinc in per kilogram of the control biomass was 69 mg. In systems containing this metal, the nostoc biomass accumulated from 453 to 3310 mg/kg of zinc. The Cu/Fe/Zn system was functional only during the first two cultivation cycles. At the end of the first cycle, the biomass accumulated 453 mg zinc/kg, which was 6.5 times more compared to the control. In the second cycle, the amount of zinc in per kilogram of the nostoc biomass increased up to 2510 mg and was 36.4 times higher compared to the control. The efficiency of zinc removal in the second cycle was approximately 100%. In the Cu/Fe/Ni/Zn system, the amount of zinc accumulated in per kilogram of biomass was 631 mg in the first cycle, 2020 mg in the second cycle, and 3310 mg in the third cycle. Zinc removal by biomass in the second and third cycles was complete.

Thus, the accumulation of copper and other metals present in the system by *Nostoc linckia* biomass significantly depended on the composition of the system and the cycle of cyanobacteria growth.

### 3.3. Change of the Biochemical Composition of Nostoc linckia Biomass, Obtained in Multimetal Systems 

**Figure 4:**
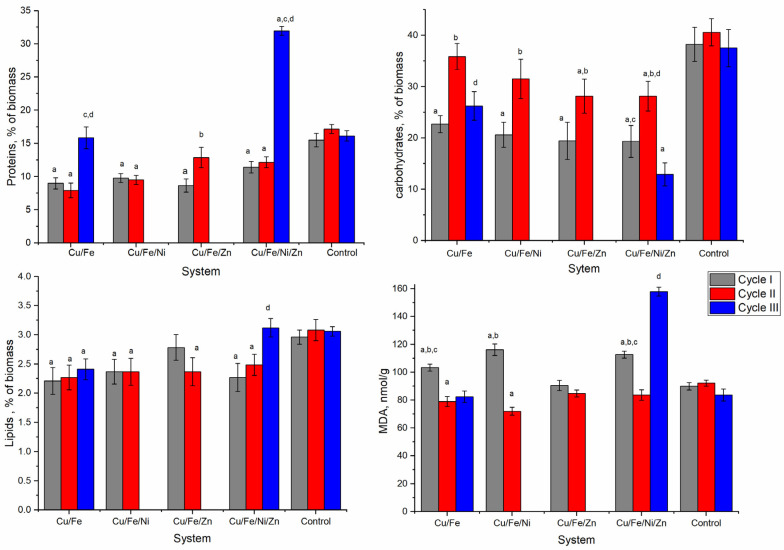
Total proteins; carbohydrates; lipids and malondialdehyde in *Nostoc linckia* biomass obtained over three cycles of cultivation in multimetallic systems (a—*p* < 0.001 for the difference between experimental and control samples, b—*p* < 0.001 for the difference between the first and second cycle, c—*p* < 0.001 for the difference between the first and third cycles, d—*p* < 0.001 for the difference between the second and third cycles).

The main structural and functional components of the *Nostoc linckia* biomass—proteins, carbohydrates, and lipids—changed significantly under the conditions of biomass cultivation in the presence of multimetal systems (Figure 4). In the main part of experimental variants, a significant decrease in the amount of total protein in the nostoc biomass was observed. 

The content of proteins in biomass obtained in the first cycle, in all four multimetal systems, varied between 8.7% and 11.4% of the dry biomass (DB) which was 26.4–42.1% lesser compared to the control (*p* < 0.001). The lowest value of this parameter was characteristic for the Cu/Fe system, and the highest—for the Cu/Fe/Ni/Zn system. The biomass in the second cultivation cycle contained 7.9–2.9% of total proteins. Only in the Cu/Fe/Zn system, a statistically significant difference (*p* < 0.005) compared to the total protein content in the nostoc biomass obtained after the first cultivation cycle was noted. 

In this case, the amount of protein increased from 8.6% ADB in the first cycle to 12.9% in the second cycle but remained 25% lower than in control biomass (*p* < 0.01). In the other three systems, there were no statistically significant differences between the protein content in the biomass after the first and second cycles of nostoc cultivation. The situation was completely different in the two multimetal systems in which the nostoc culture survived the third cycle. In the Cu/Fe system, the nostoc biomass contained 15.84% of total proteins which was equivalent to the control, while the biomass in the Cu/Fe/Ni/Zn system contained a double amount of protein compared to the control—31.9% ADB.

The content of carbohydrates in biomass grown in the polymetallic systems also changed significantly. At the end of the first cultivation cycle in all four analyzed systems, the biomass contained 40.6–49.4% fewer carbohydrates compared to the control (*p* < 0.001). In the second cycle of cultivation, nostoc biomass accumulated more carbohydrates compared to the first cycle (*p* < 0.001). Thus, in the Cu/Fe system, the content of carbohydrates reached the value of 35.83% ADB, which did not differ statistically from the carbohydrate content in the control biomass. 

In the other three systems, the content of carbohydrates in the nostoc biomass (28.1–31.48%) was higher compared to the first cultivation cycle and lower compared to the control. In the third cycle in the Cu/Fe system, the biomass of nostoc contained 26.22% ADB of carbohydrates, similar to the first cycle. In the Cu/Fe/Ni/Zn system, the content of carbohydrates in the biomass was 12.88%, which constituted 35% of the control value and was significantly lower than in the first two cultivation cycles (*p* < 0.001).

The content of lipids in nostoc also changed in the presence of metals. With the exception of the Cu/Fe/Zn system (first cycle) and Cu/Fe/Ni/Zn (third cycle), the content of lipids in experimental variants decreased by 20.0–26.2% compared to the control. In three of the four systems, at the end of the first cultivation cycle nostoc biomass contained 2.21–2.37% of lipids, which is less compared to the control with 2.96–3.08% (*p* < 0.001). Only in the Cu/Fe/Zn system, the content of lipids in the nostoc biomass at the end of the first cycle was at the level of control biomass. In the second cultivation cycle, the content of lipids in the nostoc biomass in all studied systems was lower compared to the control by 19.2–26.3% (*p* < 0.001); however, it did not differ statistically from the first cycle.

The amount of malonic dialdehyde, which is a marker of oxidative stress in the nostoc biomass, at the end of the first cultivation cycle in three systems, was significantly higher compared to the control and only in the Cu/Fe Zn system, it was at the level of the control. In the second cultivation cycle, the MDA content in biomass significantly decreased compared to the first cycle (*p* < 0.001) but was equal to (Cu/Fe/Zn and Cu/Fe/Ni/Zn systems) or slightly lower than (Cu/Fe and Cu/Fe/Ni systems) the control. In the third cycle of cultivation of biomass in the Cu/Fe system, the content of MDA was on the level of control and in the Cu/Fe/Ni/Zn system—by 88.9% higher than the control.

The most severely affected components of the nostoc biomass were phycobiliproteins, the content of which was reduced by 79.5–100.0% (Figure 4). In all analyzed systems after the first cycle of cultivation, the nostoc biomass contained small amounts of phycobiliproteins. In the second cultivation cycle, only the biomass in Cu/Fe and Cu/Fe/Ni systems contained about 2% phycobiliproteins, which constituted 18.6–19.2% of the value characteristic for the control sample. In the third cycle, only the biomass in the Cu/Fe system contained 1.16% of phycobiliproteins.

The content of β-carotene in nostoc biomass was strongly affected by heavy metals (Figure 4). The values obtained for the experimental variants constituted 32.9–55.2% of the control. The pattern of change observed in the systems was approximately the same and was expressed by the progressive decrease of the amount of β-carotene in nostoc biomass obtained in the second cultivation cycle. In the third cycle, in the nostoc biomass in the Cu/Fe system, the content of β-carotene was at the level of the second cycle, and in the Cu/Fe/Ni/Zn system—at the level of the first cycle.

**Figure 5:**
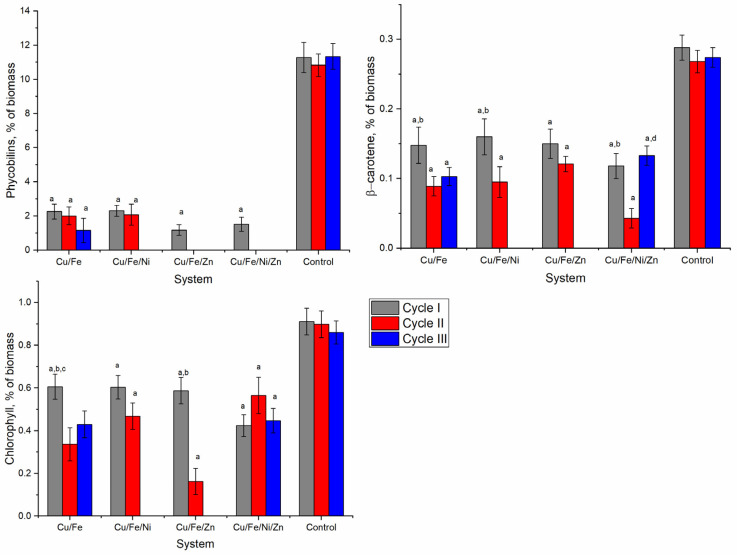
Total phycobiliproteins, chlorophyll α and β-carotene in Nostoc linckia biomass obtained during three cycles of cultivation in media containing multimetallic systems (a—*p* < 0.001 for the difference between experimental and control samples, b—*p* < 0.001 for the difference between the first and second cycle, c—*p* < 0.001 for the difference between the first and third cycles, d—*p* < 0.001 for the difference between the second and third cycles).

The content of chlorophyll was significantly reduced in all experimental samples (Figure 5). In the first cycle, the nostoc biomass accumulated in the four systems contained 33.4–53.4% less chlorophyll than the control (*p* < 0.001). In three of the studied systems (Cu/Fe, Cu/Fe/Ni, and Cu/Fe/Zn), the content of chlorophyll in the second cycle decreased compared to the first cycle and the difference relative to the control was already 48.0–81.9%. In the Cu/Fe/Ni/Zn system, the chlorophyll content in the nostoc biomass in the third cycle decreased compared to the second and returned to the level of the first cycle.

### 3.4. Antioxidant Activity of Nostoc

**Figure 6:**
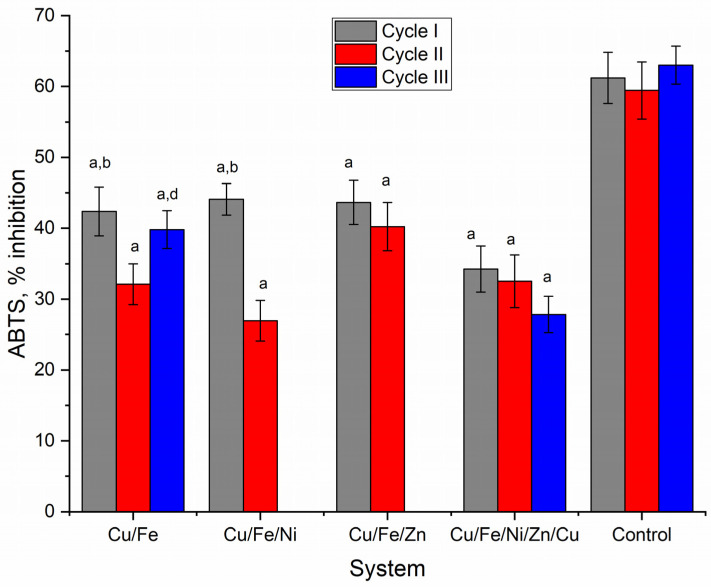
Antioxidant activity (% inhibition of ABTS^+^) of ethanolic extracts of *Nostoc linckia* biomass obtained during three cycles of cultivation in media containing multimetallic systems (a—*p* < 0.001 for the difference between experimental and control samples, b—*p* < 0.001 for the difference between the first and second cycle, d—*p* < 0.001 for the difference between the second and third cycles).

The antioxidant activity of nostoc biomass was assessed by the activity of the ethanolic extract. Significant changes in the antioxidant activity of this type of extract were observed depending on the multimetal system and the analyzed cultivation cycle (Figure 6).

The antioxidant activity values obtained in the experimental variants were significantly lower compared to the control (*p* < 0.001). At the end of the first cycle, the activity of the extracts obtained from the biomass of nostoc grown in the multimetallic systems constituted 34.2–44.1% of radical inhibition of ABTS cation, compared to 61.2% characteristic for control. The activity of the extracts obtained in the second cultivation cycle was lower compared to the first cycle in Cu/Fe and Cu/Fe/Ni systems (*p* < 0.001). 

In the other two systems, there was also a tendency of antioxidant activity decrease but the differences from the first cycle were statistically insignificant. In the third cycle, the extract obtained in the Cu/Fe system had higher activity compared to the second cycle and equal to the first cycle. In the Cu/Fe/Ni/Zn system, the activity of the ethanolic extract continuously decreased, and the difference in activity compared to the first cycle was significant (*p* < 0.001).

## 4. Discussion

The cyanobacteria *Nostoc linckia* are characterized by significant resistance to stress, a fact confirmed in our experiments. Thus, the first cycle of nostoc cultivation in multimetal systems resulted in a modest decrease (approximately by 10%) of the amount of accumulated biomass compared to the control. It seems that this culture is rather resilient to short-term stress in a manner that minimizes visible effects. The situation is different in the case of repeated stress. 

The culture subjected to the repeated action of multimetal systems experienced a significant loss of adaptive capacity and, thus, became vulnerable or even died, as was the case of the third cultivation cycle in the Cu/Fe/Ni and Cu/Fe/Zn systems. Biomass growth only for two cycles is difficult to explain since the data accumulated in present experiments are not sufficient to explain the mechanisms of toxicity of multi-metal systems. 

If, in the case of the Cu/Fe/Zn system, it can be assumed that the toxic effects appear due to overaccumulation of iron and zinc in biomass in the second cycle, then, in the Cu/Fe/Ni system, metal accumulation did not exceed the accumulation in the Cu/Fe/Ni/Zn system. It should be mentioned that, in the Cu/Fe/Zn system, the most significant reduction of the chlorophyll a content was observed (much more pronounced than in the other systems). Although it is known that nickel toxicity for cyanobacteria can be explained by its binding to sulphydryl-like groups and consequent reduction in Chl-A content [32] in Cu/Fe/Ni system, this effect was not observed. 

The effect of four analyzed multi-metal systems on cyanobacterium *Spirulina platensis* was studied as well. Even in the case of spirulina, the culture survived during three cultivation cycles in all analyzed systems, in the quaternary system biomass behaved differently from other systems. Thus, in the trinary systems content of proteins and phycobiliproteins decreased over the cycles, while in the Cu/Fe/Zn/Ni system, the most drastic reduction of these two components took place in the first cultivation cycle, after which in the following cycles an increase in their content was observed [22]. 

In the case of nostoc, in the quaternary system, a nonspecific reaction of nostoc to stress was observed, namely, a pronounced increase in protein and lipid content, which indicate a significant reorientation of biosynthetic processes. The mechanisms of this process are determined by the synergistic and antagonistic interactions between metals in the systems, but also by the specific and non-specific responses of cyanobacterial culture and are to be investigated in detail.

The most stable nostoc culture was the one grown in the Cu/Fe system, where the second and third cycles were identical according to the amount of biomass accumulated, followed by the complex Cu/Fe/Ni/Zn system, where nostoc also survived in the third cycle in contrast to the three-metallic systems.

Microorganism cultures that have the ability to bioaccumulate heavy metals behave differently in different multimetal systems, which has been observed by our group and other researchers [13,19,22,31]. This is most likely due to interactions between ions present in complex systems, and these interactions need to be studied separately in supplementary model experiments.

Copper, in concentrations above the tolerance limit, is an extremely toxic element for cyanobacteria and its accumulation is associated with multiple changes in the vital activity of cells. The biological effects on cyanobacteria are most often associated with the formation of reactive oxygen species and the destruction of DNA. At the same time, it has been established that in many microorganisms, the toxic effect of copper is associated with the replacement of iron in the Fe-S clusters of key enzymes in the cell [33]. 

The presence of iron ions in the analyzed experimental systems made it possible to supplement the needs of nostoc culture in preserving the functionality of some of the enzymes affected by this substitution. Metal-component proteins can inadequately and erroneously bind another metal instead of the required one, depending on their affinity to different metal ions. For example, according to the Irving–Williams series of bivalent metal ions, the affinity of proteins is higher for nickel than for copper and zinc but we cannot assert if this was observed in our experiments since the intracellular environment had reducing properties that could change the charge of accumulated metal ions, in particular copper and iron. 

Under normal conditions, several cellular systems, such as metal sensors, transporters, and stores ensure adequate affinity of proteins to metals and the correct binding of the metal required for the particular protein [34]. Under the conditions of high concentrations of metals, these mechanisms are disrupted and cells accumulate in excess substances that can seriously affect all metabolic processes. The accumulation of copper was directly proportional to the reduction of the productivity of the nostoc and the intensity of other destructive processes in the biomass. The content of proteins, carbohydrates, lipids, and pigments decreased in the majority of the experimental variants and denoted severe impairment of normal physiological processes. 

In the first cultivation cycle in all multimetallic systems, the content of malonic dialdehyde increased compared to the control. In this cycle, the nostoc culture resisted the influence of heavy metals and the increase of the marker of oxidative stress was absolutely predictable. In the next cycles, in the majority of experimental variants, the content of MDA decreased a process associated with the reduction of the content of lipids in biomass (MDA being one of the final products of oxidative degradation of lipids).

The content of phycobilins, chlorophyll, and β-carotene decreased significantly in all experimental variants, indicating that both the processes of photosynthesis and antioxidant protection were affected. The antioxidant activity of water extracts was negligible; therefore, it is not presented in the paper, and the ethanolic one was considerably below control. All these changes explain the drastic reduction of productivity and biomass death in two of the systems at the third cultivation cycle. As it was shown in [18], in *Nostoc muscorum*, chlorophyll synthesis was reduced by 26% in Cu(II)^-^treated cells and the content of total proteins declined by 15%. At the same time, in *Nostoc muscorum* grown in sterilized sewage wastewater, the content of chlorophyll a, carotenoids, chlorophyll a/carotenoids ratio, and protein increased with 58.8%, 50.4%, 5.6% and 34.6%, respectively, over the control culture [16]. 

A particular case in our experiments presents the third cycle in the Cu/Fe/Ni/Zn system. In this system, unlike those that contained not only copper and iron but also zinc and nickel, the nostoc culture survived one more cycle. The amount of obtained biomass was very low and its quality differed significantly—it was characterized by a doubling of the content of protein, a moderate increase in lipids content, and an increase in MDA. It was obvious a mobilization of cellular resources to confront the effects of oxidative stress.

This study conducted with the application of the edaphic cyanobacterium *Nostoc linckia* demonstrates the possibility of applying this object for the bioremediation of sites contaminated with copper in association with other heavy metals. The high metal bioaccumulation capacity, including copper, minimum environmental requirements, resistance to drought, and other weather conditions make us consider that this cyanobacterium can be considered a good candidate for the development of in situ technologies for bioremediation of copper contaminated soil.

## 5. Conclusions

Edaphic cyanobacteria *Nostoc linckia* showed a high capacity of copper, iron, nickel, and zinc bioaccumulation from multimetal systems. The accumulation efficiency of the nostoc biomass for four metals differed for each of the metal combinations. To elucidate the peculiarities of bioaccumulation of metals in multimetal systems, analogous data obtained on monometallic systems are required. Such data could be used to perform factor analysis, which, combined with a comparison of the results, would reveal common or particular aspects of the *Nostoc linckia* biomass accumulation capabilities.

At first contact with pollutants, a high tolerance to heavy metals in the environment was characteristic of the studied cyanobacterial strain. Under these conditions, the accumulation of metals in the nostoc biomass was associated with a reduction of protein, lipid, and carbohydrate content, degradation of pigments, and decrease of antioxidant capacity. Under repeated action of the pollutants, the tolerance of the culture decreased. Thus, nostoc should be explored for emergency bioremediation.

*Nostoc linckia* should be studied for the in situ bioremediation of heavy metals, including copper, contaminated sites. This requires expertise in the research of soil–metal systems.

## Figures and Tables

**Table 1:** Metal concentrations in the studied multielement systems.

Systems Designation	Metal Concentration, mg/L			
	Cu	Fe	Ni	Zn
Cu/Fe	2.5	2.0	-	-
Cu/Fe/Ni	2.5	2.0	0.5	-
Cu/Fe/Zn	2.5	2.0	-	0.5
Cu/Fe/Ni/Zn	2.5	2.0	0.5	0.5

## Data Availability

Not applicable.
